# Differential effects of acute exercise on emotional memory in men and women

**DOI:** 10.3389/fspor.2023.1062051

**Published:** 2023-05-10

**Authors:** Miyuki Nakamura, Yujiro Kawata, Masataka Hirosawa, Tsuneyoshi Ota, Nobuto Shibata

**Affiliations:** ^1^ Institute of Health and Sports Science & Medicine, Juntendo University, Chiba, Japan; ^2^ Faculty of Health and Sports Science, Juntendo University, Japan; ^3^ Graduate School of Health and Sports Science, Juntendo University, Chiba, Japan; ^4^ Juntendo Tokyo Koto Geriatric Medical Center, Tokyo, Japan

**Keywords:** exercise, emotional memory, memory, sex differences, cortisol

## Abstract

Exercise may change emotional memory, which is associated with the induction of mental disorders such as depression and anxiety. This effect of exercise may be influenced by exercise-induced cortisol release. Depending on sex, cortisol exerts differential effects on emotional memory consolidation. However, whether acute exercise and exercise-induced cortisol release have sex-dependent effects on emotional memory has not been established. Therefore, first, we aimed to determine the effects of acute exercise on emotional memory, separately for men and women, in a within-subjects design. Second, we aimed to examine whether the effects of acute exercise on emotional memory are related to the effects of exercise-induced cortisol release, separately for men and women. Sixteen healthy men and 15 healthy women were presented with positive and negative emotional images, followed by either rest or a vigorous-intensity cycling exercise condition using a within-subjects design on separate days. Salivary cortisol was measured before presenting the emotional images presentation and 20 min after each intervention. Emotional memory was assessed two days later. Vigorous-intensity exercise decreased emotional memory in women, whereas there was no change in men after rest or exercise. Cortisol levels increased after exercise intervention in both men and women, although there was no association between cortisol levels and emotional memory. These findings demonstrate that the effect of a single bout of vigorous-intensity exercise on emotional memory differs between men and women and is associated with decreased emotional memory in women.

## 1. Introduction

Emotional memory (e.g., negative and positive memory) has a variety of effects on people's emotions and moods and can sometimes act as a trigger for mental disorders. “Emotional memory” is defined as the memory of experiences that evoke an emotional reaction (1). In particular, stress-related psychiatric disorders such as depression and anxiety are characterized by a memory bias that results in a strong recall of negative information (e.g., fearful and unpleasant memories) (2, 3). Thus, it is very important to reduce negative memories and enhance positive ones. If we can establish interventions (e.g., exercise) that can alter the balance of emotional memory, we may also be able to improve the imbalance of emotional memory.

One potential mechanism that changes memory is related to stress-induced hormones (4–6). Stress elicits the secretion of related hormones via two pathways: the hypothalamic-pituitary-adrenal (HPA) axis (7) and the sympathetic-adrenal-medullary systems (8, 9); the stress hormones secreted from these two pathways are cortisol and noradrenaline, respectively (8). These stress hormones are involved in memory change (10). Especially, cortisol binds to two receptors (glucocorticoid and mineralocorticoid receptors) (4, 11, 12) in the amygdala, hippocampus, and other regions also changing memory consolidation, thus affecting memory performance (13–17). Thus, stress may influence emotional memory enhancement or reduction through cortisol.

Recent evidence reveals exercise-induced cortisol secretion rate changes. Indeed, exercise plays a role in triggering changes in cortisol levels (18, 19), where moderate-to-high-intensity exercise (at 60%–80% of maximum oxygen consumption rate) increases cortisol levels (18). Thus, exercise-induced cortisol release may affect emotional memory.

The potential effect of exercise in altering emotional memory is supported by some previous studies (Supplementary Materials S1). For example, it has been shown that a group performing a 10 min stepping exercise [at an exercise intensity of 60%–85% of the heart rate reserve (HRR)] had higher emotional memory scores two days afterward compared to those of a walking group (20). In addition, participants performing a stepping exercise (60%–85% HRR) were more likely to recall traumatic memories than participants performing a walking exercise (21). In contrast, a 15 min moderate-intensity exercise (at a speed perceived as brisk walking by the participant perception) did not affect emotional memory performance after one day, one week, or two weeks, indicating no effect of exercise on emotional memory (22). Collectively, these studies provide evidence that intense exercise, which increases cortisol levels, may change emotional memory. However, these studies have included moderate-intensity exercises in which cortisol is not elevated. Thus, to clarify whether exercise-induced cortisol affects emotional memory, it is necessary to examine the effects of high-intensity exercise, which specifically promotes cortisol release.

Sex differences in the effects of exercise-induced cortisol release and its impact on emotional memory may need to be considered because previous studies have shown that memory consolidation may differ between men and women. For example, women have higher emotional memory performance than men (23, 24). This is thought to result from neurobiological differences in response to emotional memory. To understand the underlying neural mechanism, fMRI studies have reported that women are significantly more active than men in response to emotional stimuli in brain regions (e.g., the amygdala, which induces emotional arousal, and the hippocampus, which controls memory) (24). Thus, it has been suggested that differences in sensitivity to emotional stimuli and activation of brain regions related to memory may cause the sex-related differences observed in emotional memory performance. From a biochemical standpoint, it has been suggested that cortisol is involved in the sex differences around emotional memory consolidation. The corticotropin-releasing factor (CRH), which is released from the hypothalamus to promote cortisol release, also affects the activity of the arousal-enhancing locus coeruleus (25), and the sensitivity of the locus coeruleus to CRH may be higher in women than men (26, 27). Thus, similar concentrations of CRH may increase arousal more in women than men. Increased arousal enhances emotional memories (14). Therefore, there may be sex differences in the consolidation of emotional memories, and women may enhance negative memories more than men. Indeed, one study suggested that cortisol release induced by a single bout of exercise may have differential effects on emotional memory in men and women (28). Moreover, cortisol release induced by acute psychological stress (29) and vigorous-intensity exercise intervention (28) after encoding may enhance negative memories in women. Furthermore, increased cortisol levels also enhance emotional memory (28, 29). Thus, the effect of exercise on emotional memory may differ by sex, and exercise after encoding emotional memory may enhance negative memories in women.

In summary, several studies have examined the effects of acute exercise on emotional memory, although only a few have investigated sex differences in the effect of exercise after memory encoding. In previous studies, memory tasks included spatial memory assessments (using a card game called “Memory”) (28) in which men typically perform better than women (30). Therefore, it remains unclear whether the effect of exercise on emotional memory differs by sex. In addition, despite previous studies employing a between-subjects design for comparisons, they lacked controls to adequately assess memory functions between each participant (20, 21, 28, 31). Thus, these limitations need to be addressed to examine the impact of exercise more effectively on emotional memory.

The aim of the current study was two-fold. First, we aimed to determine the effects of acute exercise on emotional memory, separately for men and women, in a within-subjects design. Second, we aimed to examine whether the effects of acute exercise on emotional memory are related to the effects of exercise-induced cortisol release, separately for men and women. Our study employed a within-subjects design to account for individual differences in each subject's memory function when examining the effects of exercise. Owing to sex differences in sensitivity to emotional stimuli and their subsequent effects on memory, women are more vulnerable to changes in emotional memory than men, especially regarding their response to stress. Therefore, we hypothesized that the effect of acute exercise on emotional memory would also differ by sex, with women enhancing negative memories more than men. As previous studies have shown that exercise-induced cortisol release is associated with emotional memory consolidation, our second hypothesis was that exercise-induced increases in cortisol may also enhance emotional memory.

## 2. Materials and methods

### 2.1. Participants

The sample size was calculated using R software. The main analysis assessed whether the effects of acute exercise on emotional memory differed according to sex. The sample size was determined using 2 (sex: male/female) × 2 (condition: rest/vigorous-intensity exercise) × 2 (emotional valence: positive/negative) three-way mixed analysis of variance (ANOVA) [*α* = 0.05, 1 − *β* = 0.80, medium effect size (*f* = 0.25)] (32), which estimated that 28 participants (14 men and 14 women) would be required to ensure adequate statistical power.

**Figure 1:**
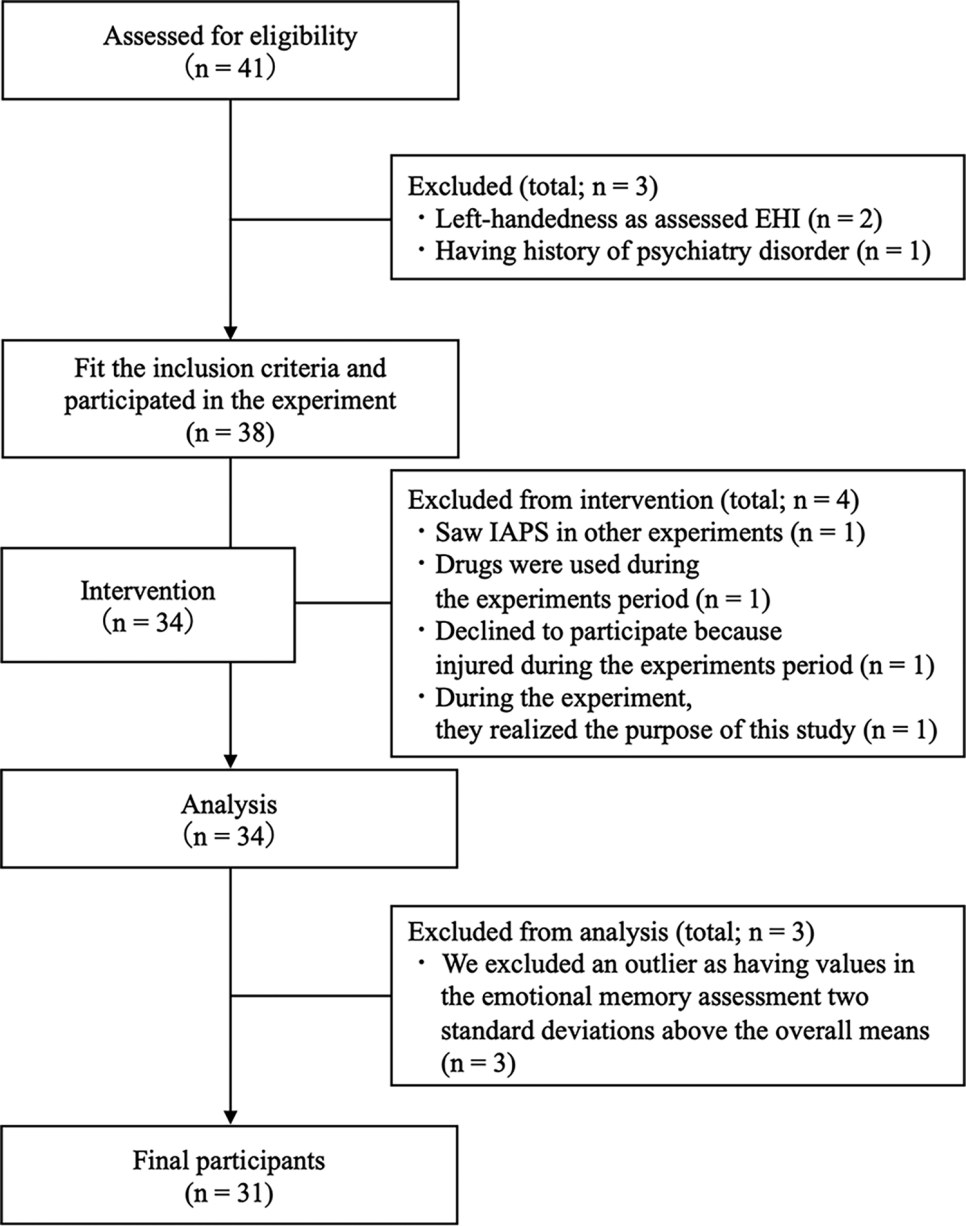
Flow diagram.

Abbreviations: EHI, Edinburgh Handedness Inventory; IAPS, International Affective Picture System.

A total of 41 healthy participants were recruited. The participants were required to fill out the Physical Activity Readiness Questionnaire (PAR-Q) (33), their medical history, and whether they had used drugs, smoked, and drunk alcohol. The inclusion criterion was no exercise at least three times a week (for more than 1 h at a time) at a higher-than-moderate intensity, assessed using the International Physical Activity Questionnaire (IPAQ) (34). The exclusion criteria comprised chronic or acute illnesses, a history of psychiatric or neurological disorders, smoking, previous drug use, previous use of hormonal contraception, and left-handedness, as assessed by the Edinburgh Handedness Inventory (35), because previous studies have reported that the dominant hand may affect memory (36, 37). Moreover, we excluded participants who had been previously exposed to the images used in the emotional memory tasks. Additionally, we excluded as outliers those that had hit rates in the emotional recognition task that were ±2 standard deviations above the mean. Finally, a total of 31 participants (16 men and 15 women; mean age = 21.84 years; standard deviation = 1.88 years) were included in this study (Figure 1). All participants were asked to refrain from drinking alcohol and caffeinated beverages, from performing physical activities the day before the experiment, and from consuming beverages (except water) and food three hours before the start of the experiment. The participants received monetary compensation of 3,000 Japanese yen.

### 2.2. Measures

#### 2.2.1. Beck depression inventory—second edition

The Beck Depression Inventory—Second Edition (BDI-II) comprises 21 self-reported items and was used to measure the participants' current depressive symptoms characteristics (38). Each item is assessed using a 4-point rating scale (0–3 based on the severity of each item). The scores for each item were used to calculate the total BDI-II score, ranging from 0 to 63. Higher scores were indicative of more severe depressive symptoms. We used the cutoff point (BDI-II score >29) described in the BDI-II manual as the criterion for exclusion of those with severe depressive symptoms (38). However, no participant in the study met this exclusion criterion.

#### 2.2.2. Depression anxiety and stress scales

Anxiety and stress were measured using the Depression Anxiety and Stress Scales (DASS) (39). The DASS includes items measuring depressive symptoms. In this study, depressive symptoms were measured using the BDI-II, and therefore the DASS depression scores were reported as supplementary information. The DASS comprises 42 items, each rated on a 4-point scale (from 0 = “Did not apply to me at all” to 3 = “Applied to me very much, or most of the time”). The scores ranged from 0 to 42 points for each factor, with higher scores being indicative of more severe symptoms. In this study, we had planned to exclude participants with DASS scores above the “severe” point, specifically, an anxiety score >15, a stress score >26, or a depression score >21. However, no participant in the study met this exclusion criterion.

#### 2.2.3. International physical activity questionnaire short form

The IPAQ short version was used to evaluate the level of physical activity, which is based on the summation of the duration (min) and frequency (days) of walking, moderate-intensity exercise, high-intensity exercise, and total physical activity (34). We used the following formulas to measure physical activities: Walking metabolic equivalent of task (MET) (min/week) = 3.3 × walking minutes × walking days; Moderate MET (min/week) = 4.0 × moderate-intensity activity minutes × moderate days; High MET (min/week) = 8.0 × high-intensity activity minutes × high-intensity days; and total physical activity MET (min/week) = sum of Walking MET + Moderate MET + High MET (min/week) scores. Higher scores were indicative of higher levels of physical activity.

### 2.3. Physiological indices

#### 2.3.1. Heart rate

HR was measured using a Polar bike computer and a Polar elastic chest strap (Polar M460, Polar H10 heart rate sensor, Polar Electro, Finland). The HR sampling rate was 1 s. Baseline HR was measured over a 3 min period after the participants had arrived at the laboratory and rested for 5 min and was defined as the mean value over the 3 min period. HR during the intervention (intervention HR) in the rest condition was measured for 20 min, from the beginning to the end of the intervention. In the vigorous-intensity exercise condition, HR was measured from the beginning of warm-up to the end of cool-down. Intervention HR data were calculated for 20 min, excluding the warm-up and cool-down periods.

#### 2.3.2. Salivary cortisol levels

Cortisol levels were measured from saliva that was collected using the passive drool method, in which drops of saliva were collected from the lower lip using a funnel (SalivaBio, Salimetrics LLC, USA). Salivary samples were immediately stored at −20°C until the assay. Salivary sampling was performed at the following time points: before presenting the emotional memory task (pre) and 20 min after the completion of each condition (post). Salivary cortisol concentrations were measured using a salivary cortisol enzyme immunoassay (EIA) kit according to the manufacturer's instructions (Cortisol EIA Kit; Salimetrics, USA).

### 2.4. Emotional memory task

The image used in the emotional memory task (the encoding task and the recognition task) were selected from the International Affective Picture System (IAPS) (40). A total of 270 images were used in the encoding and recognition tasks. One set was randomly selected for each condition and presented to the participants. We randomly set the presented order of the images based on random sampling numbers. The images were selected by comparing the mean values of valence and arousal reported in a previous study (40), to avoid differences in arousal and valence for each task. The tasks used and their average values are shown in Supplementary Materials S2–S4.

We used Presentation software version 22.0 (Neurobehavioral Systems Inc., Albany, CA, USA) to construct an emotional task, which was presented on a computer monitor, with a resolution of 1,920 × 1,080 pixels and a refresh rate of 144 Hz. The distance to the PC monitor was set at 60 cm from the participant's line of sight, with the participants seated on a chair while performing the emotional memory task.

#### 2.4.1. Encoding task

The participants were presented with 60 images (30 positive images and 30 negative images) for each condition. This task was separated into two blocks, with 30 images each, to avoid fatigue. There was a brief break lasting 2 min between the blocks. Each image was presented for 6 s, and a fixation cross was displayed for 500 ms on a computer monitor. To prevent learning effects and an excessively easy IAPS images recall, we created three image sets for the encoding tasks. Participants were instructed to push the reaction button when they saw the images.

#### 2.4.2. Recognition task

Participants were asked to return to the laboratory two days later to perform the recognition task. They were presented with 60 images (30 positive images and 30 negative images) for each condition. The recognition tasks used 30 of the images (15 positive images and 15 negative images) that were employed in the encoding task and 30 new images (15 positive images and 15 negative images) that matched the mean values of valence and arousal. Each image was presented for 3 s, and a fixation cross was displayed for 1 s on a computer monitor. Participants were required to select one of two responses (“remember” or “new”) for each image. “Remember” was defined as “I remember having seen this image 2 days prior,” and “new” was defined as “I have not seen this image 2 days prior.” Responses in the recognition task were categorized as a “hit” or a “miss.” A “hit” occurred when participants answered “remember” to an image that was indeed presented 2 days prior. A “miss” occurred when participants answered “new” to an image that had been presented 2 days prior. A third category called a “false alarm” was used if participants answered “remember” to an image that was not presented 2 days prior, and a “correct rejection” occurred if participants answered “new” to an image that was not presented 2 days prior. To prevent learning effects and an excessively easy IAPS images recall, we created three image sets for the recognition tasks.

### 2.5. Rest and vigorous-intensity exercise conditions

The present study was conducted under the following two conditions: a rest condition and a vigorous-intensity exercise condition. The rest condition required participants to sit quietly for 20 min. In the vigorous-intensity exercise condition, participants completed the exercise using a cycle ergometer (PowerMax-VIII, Combi, Tokyo, Japan). Participants started with a 1 min warm-up and then increased their HR to meet an individually calculated target HR within a period of 3 to 5 min. After reaching the target HR, participants were asked to maintain it (±5 bpm) for 2 min. Participants then commenced the exercise intervention for 20 min. They were required to sustain an HR within the target HR zone for the duration of the 20 min exercise intervention. Warm-up and cool-down required a workload of 0.5 kilopond (Kp). The pedal rate during the exercise was maintained at 50 rotations per minute. Vigorous-intensity exercise was defined using the HRR method (target HR = [(208 − (0.7 × age) − HR at rest) × 60%–85% + HR at rest]) based on the resting HR measured when participants came to the laboratory for the first time (41). The rating of perceived exertion (RPE) (42) was measured once every minute for each condition.

### 2.6. Procedure

**Figure 2:**
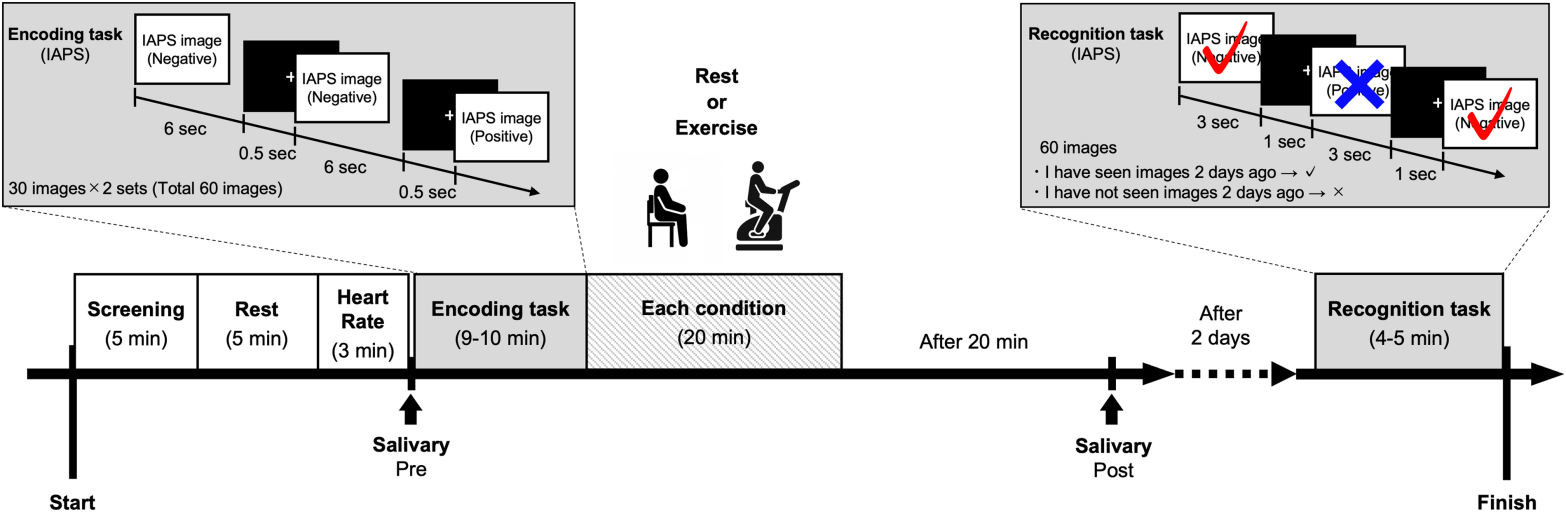
Protocol for each condition.

Abbreviations: IAPS, International Affective Picture System; Rest, rest condition; Exercise, vigorous-intensity exercise condition.

Written informed consent was obtained from all participants. The day before commencing the experiment, the participants completed questionnaires about demographic data (e.g., age and sex), and medical, drug use, smoking, alcohol consumption histories, the PAR-Q, BDI-II, DASS, and IPAQ (short version) online survey. On Day 1, the height, weight, body mass index (BMI), and HR at rest (First HR) of each participant were determined as baseline measurements. The participants then completed the encoding task and each condition. Participants were required to return to the laboratory 2 days after the Day 1 experiment to perform the recognition task on Day 2. The procedure is illustrated in Figure 2. The present study was conducted under two conditions (rest and exercise). The experiments were conducted with intervals of at least 1 week between each condition to avoid the influence of each condition. Additionally, the order of these conditions and the emotional tasks was counterbalanced and randomly assigned. The experiment was conducted between 13:00 and 18:30. We did not inform participants about the purpose of the memory task to avoid any confounding toward memory, and we used the deception “This study aims to examine the effects of exercise on visual stimulation and mood” for participants. A debriefing session was held after the completion of all conditions. This study was approved by the research ethics committee at the first author's affiliated organization.

### 2.7. Analysis of salivary cortisol

Salivary samples were immediately stored at −20°C until the time of the assay. According to the manufacturer's protocol, cortisol concentrations of all samples were determined using a commercially available salivary cortisol EIA kit (Cortisol EIA Kit; Salimetrics, USA). The inter-assay variability was 8.1%–12.4%, and the intra-assay of variability was 7.0%–11.6%. We used the pre- and post-values of the cortisol measurements for each condition, and the cortisol responses were calculated as the change in salivary cortisol levels (cortisol Δ) and the rate of change in salivary cortisol levels (cortisol Δ%). Cortisol Δ was defined as the “cortisol level 20 min after intervention (post)  − cortisol level before intervention (pre),” and cortisol Δ% was calculated as “(cortisol level 20 min after intervention [post] − cortisol level before intervention [pre]/cortisol level before intervention [pre] × 100).”

### 2.8. Analysis of recognition task data

The recognition task score was calculated using the hit rate, false alarm rate, and sensitivity (*d*′; *d*-prime). This index was calculated for each emotional valence. The hit rate was calculated using the following formula: Hit rate = hit/(hit + miss). The false alarm rate was calculated using the following formula: False alarm rate = false alarm/(false alarm + correct rejection). The sensitivity (*d*′) was calculated by transforming the hit rate and false alarm rate into a *Z* score and using the following formula: *d*′ = *Z* (hit rate) − *Z* (false alarm rate). In the case of Hit = 1 or False alarm = 0, we employed the 1/(2*N*) rule (43).

### 2.9. Data analysis

In this study, parametric tests were used to analyze all normally distributed data, whereas non-parametric tests were used to analyze non-normally distributed data.

First, demographic (age and IPAQ) and some physical data (First HR) were analyzed using Mann–Whitney *U* tests and *t*-tests to examine whether there were sex-dependent differences. Other physical data (weight and BMI) were analyzed using one-way repeated measures ANOVAs or Freedman tests to examine differences among conditions (baseline, rest condition, and exercise condition).

A 2 (sex: male/female) × 2 (condition: rest/vigorous-intensity exercise) × 2 (time; HR: baseline/intervention; cortisol: pre/post) three-way mixed ANOVA was used to assess the changes in HR and salivary cortisol levels in each condition. RPE data were analyzed using separate 2 (sex: male/female) × 2 (condition: rest/vigorous-intensity exercise) ANOVAs.

Next, to examine the effects of exercise intensity on physiological responses in men and women, HR and cortisol responses were analyzed using unpaired *t*-tests.

Subsequently, to examine sex-dependent effects of acute exercise on emotional memory (hit rate, false alarm rate, and *d*′), recognition task data were analyzed using 2 (sex: male/female) × 2 (condition: rest/vigorous-intensity exercise) × 2 (emotional valence: positive/negative) three-way mixed ANOVAs. Post-hoc testing was performed using the Bonferroni method.

Finally, we assessed the correlation between cortisol responses (cortisol Δ and cortisol Δ%) and emotional memory (hit rate and false alarm rate) for men and women and for each condition separately, using Spearman's rank correlation coefficients.

The statistical analyses were performed using IBM Statistical Package for the Social Sciences (SPSS) Statistics for Mac, version 27. Statistical significance was defined as *p* < 0.05 for all analyses.

## 3. Results

### 3.1. Participant characteristics

The participants' demographic, psychometric, and physical data are shown in Tables 1, 2. There were no significant differences in demographic data (age and IPAQ) data or physical data such as First HR between men and women (*p*s > 0.05) (see Table 1), and other physical data (weight and BMI) did not differ between conditions (*p*s > 0.05) (see Table 2).

**Table 1.** Demographic data and physiological characteristics of the participants.

		Male (*n* = 16)		Female (*n* = 15)		Total (*n* = 31)	
Age (years)		22.31	(1.96)	21.33	(1.72)	21.84	(1.88)
BDI-Ⅱ score		6.94	(5.60)	6.93	(6.15)	6.94	(5.77)
DASS	Depression	3.81	(4.64)	3.93	(5.34)	3.87	(4.90)
	Anxiety	3.75	(2.91)	3.53	(2.53)	3.65	(2.69)
	Stress	4.06	(3.77)	2.93	(3.17)	3.52	(3.48)
IPAQ	Walking (MET-min/week)	1,133.34	(1,427.91)	957.00	(828.86)	1,048.02	(1,161.07)
	Moderate (MET-min/week)	402.50	(600.88)	181.33	(304.58)	295.48	(486.26)
	High (MET-min/week)	617.00	(942.65)	208.00	(325.38)	419.10	(732.72)
	Total physical activity (MET-min/week)	2,152.84	(2,132.62)	1,346.33	(1,073.52)	1,762.60	(1,726.18)
Height (cm)		173.76	(5.12)	159.35	(6.38)	166.79	(9.25)
Weight (kg)		69.04	(8.41)	55.32	(6.47)	62.40	(10.18)
BMI (kg/m^2^)		22.84	(2.48)	21.69	(1.69)	22.29	(2.18)
First HR (BPM)		75.49	(9.02)	82.43	(11.61)	78.85	(10.77)

Data are presented as mean (standard deviations). BDI-II, Beck Depression Inventory-Second Edition; DASS, Depression Anxiety Stress Scale; IPAQ, International Physical Activity Questionnaire; MET, metabolic equivalent of task; BMI, body mass index; HR, heart rate; First HR, resting heart rate for setting exercise intensity; BPM, beats per minute.

**Table 2.** Physiological characteristics in each condition divided by sex.

	Male (*n* = 16)				Female (*n* = 15)				Total (*n* = 31)			
	Rest condition		Exercise condition		Rest condition		Exercise condition		Rest condition		Exercise condition	
Weight (kg)	69.20	(8.97)	69.03	(8.45)	55.03	(6.43)	54.99	(6.63)	62.34	(10.55)	62.24	(10.35)
Body Mass Index (kg/m^2^)	22.89	(2.63)	22.83	(2.47)	21.56	(1.64)	21.55	(1.70)	22.25	(2.27)	22.21	(2.19)
Baseline HR (BPM)	72.84	(9.53)	74.19	(13.23)	79.72	(12.14)	83.69	(15.21)	76.17	(11.76)	78.79	(14.79)
Intervention HR (BPM)	69.67	(6.56)	164.20	(10.38)	75.12	(11.12)	170.50	(4.81)	72.31	(9.32)	167.25	(8.65)
RPE	6.53	(0.86)	16.08	(1.89)	6.37	(0.56)	16.20	(2.07)	6.45	(0.73)	16.13	(1.94)
Baseline CORT (µg/dL)	0.19	(0.09)	0.25	(0.10)	0.30	(0.16)	0.28	(0.14)	0.24	(0.14)	0.26	(0.12)
Post CORT (µg/dL)	0.17	(0.09)	0.51	(0.34)	0.17	(0.06)	0.66	(0.53)	0.17	(0.07)	0.58	(0.43)
Cortisol Δ	−0.03	(0.10)	0.26	(0.36)	−0.13	(0.14)	0.38	(0.54)	−0.08	(0.13)	0.32	(0.45)
Cortisol Δ%	−6.72	(41.18)	149.97	(185.15)	−34.66	(28.38)	170.56	(299.78)	−20.23	(37.75)	159.93	(243.29)
	Male				Female				*t*		*p*-value	*d*
	Mean		SD		Mean		SD					
HR Δ’	90.01		12.34		86.91		12.81		0.71		0.48	0.25
HR Δ’%	126.46		33.24		109.35		33.84		1.42		0.17	0.51
Cortisol Δ’	0.26		0.36		0.38		0.54		−0.69		0.50	0.26
Cortisol Δ’%	149.97		185.15		170.56		299.78		−0.23		0.82	0.08

Data are presented as mean (standard deviations). Exercise condition, vigorous-intensity exercise condition; HR, heart rate; BPM, beats per minute; RPE, rated perceived exertion; CORT, salivary cortisol.

HR Δ′ was defined and calculated as “(exercise condition of intervention HR − exercise condition of baseline HR) − (rest condition of intervention HR − rest condition of baseline HR),” and HR Δ′% was defined and calculated as “((exercise condition of intervention HR − exercise condition of baseline HR) − (rest condition of intervention HR − rest condition of baseline HR))/(rest condition of intervention HR − rest condition of baseline HR) × 100.” Cortisol Δ′ was defined and calculated as “(cortisol Δ of exercise condition − cortisol Δ of rest condition),” and cortisol Δ′% was defined and calculated as “((cortisol Δ of exercise condition − cortisol Δ of rest condition)/cortisol Δ of rest condition) × 100.”

### 3.2. Manipulation check

#### 3.2.1. Heart rate

The baseline HR and intervention HR for each condition are presented in Table 2. A 2 (sex: male/female) × 2 (condition: rest/vigorous-intensity exercise) × 2 (time: baseline/intervention) three-way mixed ANOVA revealed a significant condition × time interaction (*F*_(1, 29)_ = 1,663.36, *p* < 0.001, $\eta_{p}^{2}$ = 0.983), although no significant sex × condition (*F*_(1, 29)_ = 0.47, *p* = 0.50, $\eta_{p}^{2}$ = 0.016), sex × time (*F*_(1, 29)_ = 0.82, *p* = 0.37, $\eta_{p}^{2}$ = 0.027), or Sex × Condition × Time (*F*_(1, 30)_ = 0.15, *p* = 0.70, $\eta_{p}^{2}$ = 0.005) interactions were observed. The simple main effect assessment using the Bonferroni post-hoc test revealed a significant difference in time for both the rest condition (baseline > intervention, *p* < 0.001, $\eta_{p}^{2}$ = 0.414) and the vigorous-intensity exercise condition (baseline < intervention, *p* < 0.001, $\eta_{p}^{2}$ = 0.981). At the time of intervention, there was a significant difference concerning condition (rest condition < vigorous-intensity exercise condition, *p* < 0.001, $\eta_{p}^{2}$ = 0.993), which was not observed at baseline (*p* = 0.17, $\eta_{p}^{2}$ = 0.063). These findings confirmed that the rest condition was effective in decreasing participant HR, whereas the vigorous-intensity exercise intervention was effective in increasing participant HR.

#### 3.2.2. RPE

A separate 2 (sex: male/female) × 2 (condition: rest/vigorous-intensity exercise) ANOVA revealed a significant main effect of condition (*F*_(1, 29)_ = 758.26, *p* < 0.001, $\eta_{p}^{2}$ = 0.963), although there was no significant main effect of sex (*F*_(1, 29)_ = 0.003, *p* = 0.96, $\eta_{p}^{2}$ < . 001) or an interaction effect (*F*_(1, 29)_ = 0.17, *p* = 0.68, $\eta_{p}^{2}$ = 0.006). These results indicated that the RPE was significantly higher in the vigorous-intensity exercise condition than in the rest condition.

#### 3.2.3. Salivary cortisol

**Figure 3:**
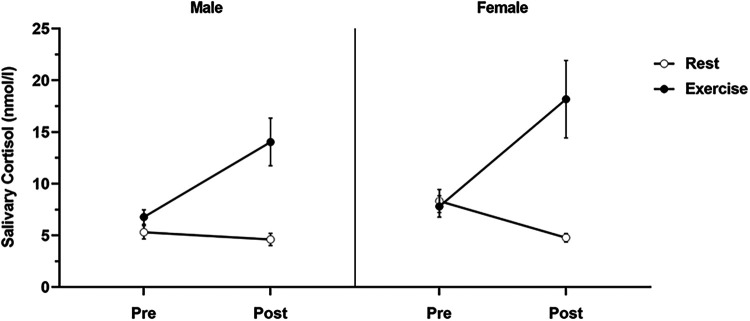
Salivary cortisol levels for each condition by sex. Salivary cortisol levels for each condition were determined before the intervention (pre) and 20 min after the intervention (post). Data are presented as mean ± standard error of the mean.

Abbreviations: Rest, rest condition; Exercise, vigorous-intensity exercise condition.

Figure 3 displays the salivary cortisol data pre- and post-intervention for each condition in both men and women. A 2 (sex: male/female) × 2 (condition: rest/vigorous-intensity exercise) × 2 (time: pre/post) three-way mixed ANOVA revealed a significant condition × time interaction (*F*_(1, 29)_ = 22.76, *p* < 0.001, $\eta_{p}^{2}$ = 0.440). However, no significant interaction was found for sex × condition (*F*_(1, 29)_ = 0.16, *p* = 0.69, $\eta_{p}^{2}$ = 0.005), sex × time (*F*_(1, 29)_ = 0.003, *p* = 0.96, $\eta_{p}^{2}$ < . 001), or sex × condition × time (*F*_(1, 26)_ = 1.69, *p* = 0.20, $\eta_{p}^{2}$ = 0.055). The simple main effect assessment using the Bonferroni post-hoc test revealed a significant difference regarding time for the rest condition (pre > post, *p* = 0.001, $\eta_{p}^{2}$ = 0.308) and the exercise condition (pre < post, *p* < 0.001, $\eta_{p}^{2}$ = 0.344). There was a significant difference between conditions after exercise (rest condition < vigorous-intensity exercise condition, *p* < 0.001, $\eta_{p}^{2}$ = 0.468), but not before exercise (*p* = 0.55, $\eta_{p}^{2}$ = 0.012). Cortisol levels were elevated post-intervention compared with pre-intervention levels in the vigorous-intensity exercise condition.

#### 3.2.4. Effects of exercise intensity on physiological responses in men and women

We examined the effects of exercise intensity on physiological responses in men and women (see Table 2). The results revealed no significant differences between men and women in HR Δ′ (*t* (29) = 0.71, *p* = 0.48, *d* = 0.25), HR Δ′% (*t* (29) = 1.42, *p* = 0.17, *d* = 0.51), cortisol Δ′ (*t* (29) =  −0.69, *p* = 0.50, *d* = 0.26), and cortisol Δ′ (*t* (29) =  −0.23, *p* = 0.82, *d* = 0.08).

### 3.3. Effects of acute exercise on emotional memory recognition performance

#### 3.3.1. Effects of acute exercise on the hit rate in the emotional memory task

**Figure 4:**
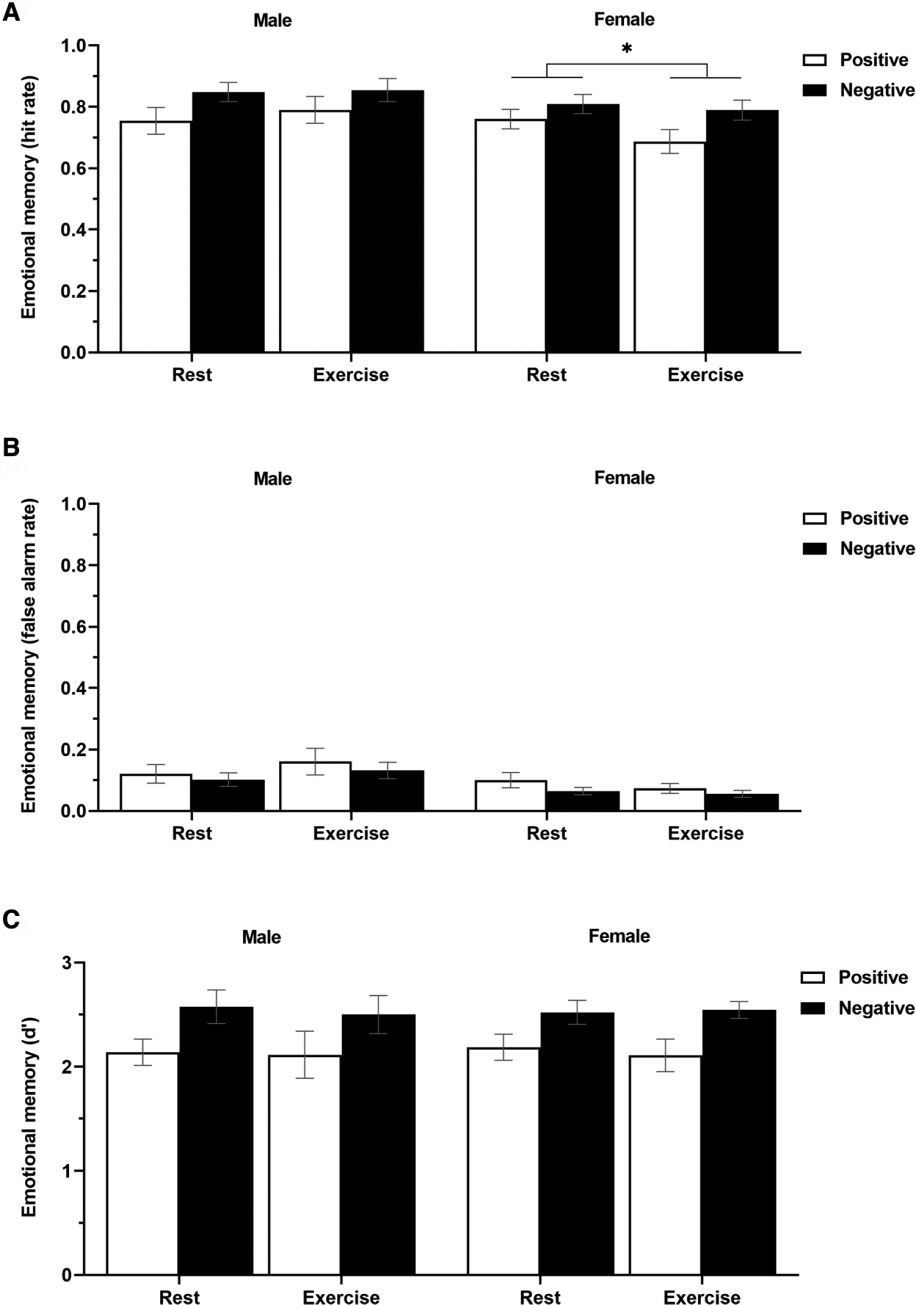
The hit rate (**A**), false alarm rate (**B**), and sensitivity (**C**) in the emotional memory task (positive and negative) in men and women for each condition (rest and exercise). Data are presented as mean ± standard error of the mean.

Abbreviations: Rest, rest condition; Exercise, vigorous-intensity exercise condition; Positive, positive emotional memory; Negative, Negative emotional memory.

Figure 4A and Table 3 display the effects of acute exercise on the hit rate in the emotional memory task. A 2 (sex: male/female) × 2 (condition: rest/vigorous-intensity exercise) × 2 (emotional valence: positive/negative) three-way mixed ANOVA revealed a significant main effect of emotional valence (*F*_(1, 29)_ = 19.65, *p* < 0.001, $\eta_{p}^{2}$ = 0.404), or sex × condition interaction (*F*_(1, 29)_ = 4.61, *p* = 0.04, $\eta_{p}^{2}$ = 0.137), although there were no significant main effects of sex (*F*_(1, 29)_ = 1.34, *p* = 0.26, $\eta_{p}^{2}$ = 0.044), or condition (*F*_(1, 29)_ = 0.68, *p* = 0.42, $\eta_{p}^{2}$ = 0.023), or interactions between sex × emotional valence (*F*_(1, 29)_ = 0.01, *p* = 0.92, $\eta_{p}^{2}$ < . 001), or condition × emotional valence (*F*_(1, 29)_ = 0.13, *p* = 0.72, $\eta_{p}^{2}$ = 0.005), or sex × condition × emotional valence (*F*_(1, 29)_ = 1.53, *p* = 0.23, $\eta_{p}^{2}$ = 0.050). The Bonferroni post-hoc tests revealed that emotional memory performance was significantly lower in women in the exercise condition than in the rest condition (rest > vigorous-intensity exercise, *p* < 0.05, $\eta_{p}^{2}$ = 0.128); however, there was no significant difference between conditions in men (*p* = 0.35, $\eta_{p}^{2}$ = 0.030). There were no significant sex differences in either the rest condition (*p* = 0.71, $\eta_{p}^{2}$ = 0.005) or the vigorous-intensity exercise condition (*p* = 0.09, $\eta_{p}^{2}$ = 0.095). These findings suggested that vigorous-intensity exercise tended to be associated with decreased emotional memory in women.

**Table 3.** Emotional memory in each condition divided by sex.

		Male (*n* = 16)						Female (*n* = 15)				
		Rest condition			Exercise condition		Rest condition			Exercise condition		
Valence												
Positive	Hit rate	0.75		(0.17)	0.79	(0.17)	0.76		(0.12)	0.68		(0.15)
	False alarm rate	0.12		(0.12)	0.16	(0.17)	0.10		(0.10)	0.07		(0.06)
	*d’*	2.14		(0.50)	2.12	(0.91)	2.19		(0.48)	2.11		(0.60)
Negative	Hit rate	0.85		(0.13)	0.85	(0.15)	0.81		(0.12)	0.79		(0.17)
	False alarm rate	0.10		(0.09)	0.16	(0.17)	0.06		(0.05)	0.06		(0.04)
	*d’*	2.58		(0.65)	2.50	(0.73)	2.52		(0.45)	2.55		(0.31)
		Hit rate					False alarm rate			*d’*		
		*F*	*p*	*η^2^p*	Post-hoc test		*F*	*p*	η^2^p	*F*	*p*	*η^2^p*
Sex		1.34	0.26	0.04			4.04	0.05	0.12	<0.01	0.96	<0.001
Condition		0.66	0.42	0.02			0.24	0.63	0.01	0.16	0.69	0.01
Emotional valence		19.65	<0.001	0.04			3.99	0.06	0.12	24.91	<.001	0.46
Sex × Condition		4.61	0.04	0.14	Women (Rest > exercise) *p* < .05		2.37	0.14	0.08	0.01	0.91	<0.001
Sex × Emotional valence		0.01	0.92	<0.001			0.01	0.91	<0.001	0.03	0.87	<0.01
Condition × Emotional valence		0.13	0.72	<0.01			0.03	0.86	0.001	0.02	0.88	<0.01
Sex × Condition × Emotional valence		1.53	0.23	0.05			0.42	0.52	0.01	0.21	0.65	0.01

Data are presented as mean (standard deviations). Exercise condition, vigorous-intensity exercise condition; Sex, male and female; Condition, rest and vigorous-intensity exercise condition; Emotional valence, positive and negative.

#### 3.3.2. Effects of acute exercise on the false alarm rate in the emotional memory task

Figure 4B and Table 3 display the effects of acute exercise on the false alarm rate in the emotional memory task. A 2 (sex: male/female) × 2 (condition: rest/vigorous-intensity exercise) × 2 (emotional valence: positive/negative) three-way mixed ANOVA revealed no significant main effect of sex (*F*_(1, 29)_ = 4.04, *p* = 0.05, $\eta_{p}^{2}$ = 0.122), condition (*F*_(1, 29)_ = 0.24, *p* = 0.63, $\eta_{p}^{2}$ = 0.008), or emotional valence (*F*_(1, 29)_ = 3.99, *p* = 0.06, $\eta_{p}^{2}$ = 0.121), or a sex × condition (*F*_(1, 29)_ = 2.37, *p* = 0.13, $\eta_{p}^{2}$ = 0.075), sex × emotional valence (*F*_(1, 29)_ = 0.01, *p* = 0.91, $\eta_{p}^{2}$ < . 001), condition × emotional valence (*F*_(1, 29)_ = 0.03, *p* = 0.86, $\eta_{p}^{2}$ = 0.001), or sex × condition × emotional valence (*F*_(1, 29)_ = 0.42, *p* = 0.52, $\eta_{p}^{2}$ = 0.014) interaction effect.

#### 3.3.3. Effects of acute exercise on sensitivity (*d*′) in the emotional memory task

Figure 4C and Table 3 display the effects of acute exercise on *d*′ in the emotional memory task. A 2 (sex: male/female) × 2 (condition: rest/vigorous-intensity exercise) × 2 (emotional valence: positive/negative) three-way mixed ANOVA revealed a significant main effect of emotional valence (*F*_(1, 29)_ = 24.91, *p* < 0.001, $\eta_{p}^{2}$ = 0.462). However, there was no significant main effect of sex (*F*_(1, 29)_ = 0.003, *p* = 0.96, $\eta_{p}^{2}$ < . 001), or condition (*F*_(1, 29)_ = 0.16, *p* = 0.69, $\eta_{p}^{2}$ = 0.006), or a sex × condition (*F*_(1, 29) _= 0.01, *p* = 0.91, $\eta_{p}^{2}$ < . 001), sex × emotional valence (*F*_(1, 29)_ = 0.03, *p* = 0.87, $\eta_{p}^{2}$ = 0.001), condition × emotional valence (*F*_(1, 29)_ = 0.02, *p* = 0.88, $\eta_{p}^{2}$ = 0.001), or sex × condition × emotional valence (*F*_(1, 29)_ = 0.21, *p* = 0.65, $\eta_{p}^{2}$ = 0.007) interaction effect.

### 3.4. Correlation between cortisol levels and emotional memory recognition performance

We further examined the correlation between salivary cortisol responses (cortisol Δ, cortisol Δ%) and emotional memory recognition performance (hit rate and false alarm rate) separately for men and women (Table 4). There was no significant correlation between cortisol levels and the other measures of emotional memory recognition performance in men or women (*p* > 0.05).

**Table 4:** Relationships between salivary cortisol levels and emotional memory performance divided by sex.

		Male				Female			
		Cortisol Δ		Cortisol Δ%		Cortisol Δ		Cortisol Δ%	
		*r*	*p*-value	*r*	*p*-value	*r*	*p*-value	*r*	*p*-value
Hit rate									
Rest condition	Positive	.44	.09	.43	.10	−.09	.75	−.11	.69
	Negative	.30	.27	.30	.26	.38	.17	.33	.23
Exercise condition	Positive	.04	.89	−.03	.90	.11	.71	.16	.58
	Negative	−.24	.36	−.29	.28	−.04	.88	−.02	.94
False alarm rate									
Rest condition	Positive	.03	.92	−.05	.87	.32	.25	.23	.40
	Negative	.03	.91	.06	.83	−.24	.39	−.33	.24
Exercise condition	Positive	.23	.38	.12	.65	−25	.37	−.27	.33
	Negative	.09	.74	−.05	.84	.24	.40	.31	.27

Exercise condition, vigorous-intensity exercise condition.

## 4. Discussion

The present study aimed to determine the effects of acute exercise on emotional memory, separately for men and women, in a within-subjects design. Second, we aimed to examine whether the effects of acute exercise on emotional memory are related to the effects of exercise-induced cortisol release, separately for men and women. Our results showed that the hit rate in the emotional memory task was decreased to a greater extent in the vigorous-intensity exercise condition than in the rest condition in women, and there was no difference in the false alarm rate and *d*′ between conditions in either men or women. Thus, we showed that acute vigorous-intensity exercise decreased emotional memory in women but not in men, and the exercise-induced increase in cortisol was not associated with emotional memory in either the men or women participants.

First, our results support our hypothesis that the effect of acute vigorous-intensity exercise on emotional memory would differ by sex. These results may be related to sex differences in the consolidation of emotional memories as shown by previous studies (23, 24). In particular, it has been reported that women significantly activate brain regions such as the amygdala (44, 45) and hippocampus in response to emotional stimuli more than men (24). Exercise increases activation in these brain regions (46). Thus, the effects of exercise may have facilitated responses to emotional stimuli, contributing to the sex differences.

However, the results did not support the hypothesis that women enhance emotional memory more than men. This may be because the activated HPA axis and increased arousal due to acute vigorous-intensity exercise affected emotional memory. During stress and arousal, the HPA system, which mediates cortisol responses, increases arousal. These changes occur in response to the corticotropin releasing factor (CRF); this factor is released by the hypothalamus and induces cortisol release through the HPA system, which increases the activation of the noradrenergic locus coeruleus (LC neurons) (25, 27, 47). The LC neurons increase arousal (48, 49). A low dose of CRF that has no effect on LC neuron activation in males does increase LC neuron activation in females (26, 27); in other words, women have higher arousal than men at similar CRH doses. Increasing arousal may enhance (13) or decrease emotional memory. Albeit without certainty, very high levels of cortisol may reduce emotional memory, as observed in this study. However, the mechanism by which transient exercise affects emotional memory is unclear, and the results of a previous study (28) have been inconsistent. Thus, further research is required in this area.

Next, our results did not support the hypothesis that there would be an association between exercise-induced cortisol levels and emotional memory in men and women. Cortisol has previously been found to be implicated in memory changes (50, 51). Exercise-induced increase in cortisol is associated with emotional memory (28). In contrast, stress-induced cortisol levels were shown not to be related to emotional memory (16, 52), while pharmacologically administered cortisol can be associated with memory performance, in an inverted U-shaped relationship (53). Cortisol may not have a linear effect on emotional memory, which could be a possible explanation for the results of the present study. One reason for a lack of a linear relationship between cortisol and emotional memory may be related to the interaction between cortisol and the hippocampus, which controls memory (54). The hippocampus has glucocorticoid and mineralocorticoid receptors (4, 11, 12). Glucocorticoids bind directly to mineralocorticoid receptors (55) and other glucocorticoid receptors (56, 57); this affects memory function via receptor activation (5). However, these two receptor types have different affinities for cortisol (58, 59), and it has been suggested that the occupancy of each receptor may determine memory enhancement or decrease (4, 12, 10, 60). Hence why cortisol secretion may exhibit an inverted-U-shaped dose-response relationship with emotional memory. In fact, a previous study examining the association between cortisol responsiveness and memory revealed that individuals with higher stress-induced cortisol increases had greater memory decreases than individuals with more modest cortisol responses (61). Cortisol may not have had a direct effect on emotional memory, which may have led to the results of this study (no association between cortisol and emotional memory). Thus, future studies should examine exercise-induced cortisol reactivity in more detail, considering sex differences.

Finally, our study had several limitations. First, we did not control for activities performed from the time of the intervention until the day of the emotional memory test two days later. We instructed participants not to exercise during this period and checked their self-reported activity. However, we did not measure the amount of physical activity using wearable monitors. Therefore, the results could have been affected by any physical activity performed but not reported during this period. Next, our study excluded participants using hormonal contraceptives and did not take into account menstrual cycle for the female participants in each condition. The menstrual cycle may affect cortisol secretion as a result of changes in sex hormones secretion (62). Furthermore, it has been reported that the menstrual cycle affects emotional memory (63). Therefore, an experiment designed to control for the menstrual cycle would be needed to examine the association between cortisol and emotional memory more precisely in women. Next, our sample size was small to examine the association between cortisol and emotional memory. Examining the effects in a larger sample would provide stronger evidence for the association between cortisol and emotional memory. Next, the present study did not measure motivation to perform the emotional memory task, which could have affected the results. In future studies, motivation to perform the emotional memory task should be measured using a questionnaire. Finally, our study did not compare non-emotional (e.g., neutral memory) and emotional memories (e.g., negative and positive memories) regarding the effects of exercise on emotional memory. Thus, exercise may affect not only emotional but also episodic memory (which includes emotional memory). Future studies shall examine the effects of exercise on both non-emotional and emotional memory.

## 5. Conclusion

In conclusion, our study confirmed that vigorous-intensity exercise affects emotional memory in a sex-specific manner, as it decreases emotional memory in women. The exercise-induced increase in cortisol was not related to measures of emotional memory. This study provides the first evidence of the effect of exercise on emotional memory using a within-subjects design. However, owing to the above-mentioned limitations, this study's results must be considered preliminary. Consequently, to develop exercise programs for the prevention and treatment of mental disorders such as depression and anxiety, it is necessary to consider timing, intensity, and frequency of exercise, and sex differences to examine their effects on emotional memory.

## Data Availability

The raw data supporting the conclusions of this article will be made available by the authors, without undue reservation.
