# Supplementary material for: Differential effects of acute exercise on emotional memory in men and women
Source: Front Sports Act Living. 2023 May 10;5:1062051. doi: 10.3389/fspor.2023.1062051 (PMC10208400; doi:10.3389/fspor.2023.1062051)
Supplement: Supplementary file 1 [file Datasheet1.docx]

Supplementary Material

Differential effects of acute exercise on emotional memory in men and women

Miyuki Nakamura, Yujiro Kawata^*^, Masataka Hirosawa, Tsuneyoshi Ota, Nobuto Shibata

*** Correspondence:**Yujiro Kawata, Ph.D.
[yuukawa@juntendo.ac.jp](mailto:yuukawa@juntendo.ac.jp)

Contents

| Supplemental material | Page |
| --- | --- |
| Supplementary material 1. Summary of previous studies on the effects of acute exercise on emotional memory | 2 |
| Supplementary material 2. IAPS pictures number (Encoding task) | 4 |
| Supplementary material 3. IAPS pictures number (Recognition task) | 5 |
| Supplementary material 4. Valence and arousal rating of IAPS images | 6 |
| Supplementary material 5. Comparing positive and negative images of valence and arousal rating | 7 |

Supplementary material 1. Summary of previous studies on the effects of acute exercise on emotional memory

| **Authors** | **Participants** | **Study design** | **Each group type** | **Memory task** | | | **Results** |
| --- | --- | --- | --- | --- | --- | --- | --- |
|  |  |  |  | **Encoding task** | **Timing** | **Recall or Recognition task** |  |
| Keyan et al., 2017 (20) | 62 healthy adults (20 males, 42 females) aged 18–36 years | Experimental: between-subject, control group vs exercise group | Control: slow walking  Exercise: stepping exercise (60–85% HRR) | IAPS (40), 36 images (positive 18, negative 18) | An encoding task was performed after each intervention. | A free recall task was performed 2 days after the encoding task. | Compared with the control group, participants in the exercise group recalled more emotional images. |
| Keyan et al., 2017 (21) | 49 healthy university students (16 males, 33 females) aged 18–29 years | Experimental: between-subject, control group vs exercise group | Control: easy walking  Exercise: stepping exercise (50–85% HRR) | Car accident film | Each intervention was performed after watching the car accident film (encoding task). | Two days after the experiment participants were assessed for both intrusive memories and voluntary memories of the car accident film details. | Compared with the control group, the exercise group reported more intrusive memories, but not more voluntarily recalled memories, of the car accident film. |
| Wade et al., 2018 (22) | 34 healthy adults (17 males, 17 females) aged 18–35 years | Experimental: between-subject, control group vs exercise group | Control: no exercise  Exercise: walking on a treadmill at a brisk intensity | IAPS (40), 50 images | An encoding task was performed after each intervention. | The recognition task was performed 1, 7, and 14 days after each intervention. | Recognition memory decreased over time in both groups.  There was no difference in recognition memory between the control and exercise groups. |
| Jentsch et al., 2020 (28) | 48 healthy adults (22 males, 26 females) aged 18–35 years | Experimental: between-subject, control group vs exercise group | Control: easy walking  Exercise: treadmill exercise (85% HRR) | A computerized version of the card game “Memory” (This task uses IAPS). | Each intervention was performed after the encoding task. | A memory retention test was performed 2 days after the encoding task. | In males, recall of positive items was enhanced.  In females, recall of negative items was enhanced. |

Abbreviations: HRR, heart rate reserve ([heart rate max − heart rate at rest] + heart rate at rest); IAPS, International Affective Picture System heart rate

Supplementary Data

Supplementary material 2. IAPS pictures number (Encoding task)

| Set 1 | |  | Set 2 | |  | Set 3 | |
| --- | --- | --- | --- | --- | --- | --- | --- |
| 8191 | 8400 |  | 8118 | 6250.2 |  | 9800 | 1645 |
| 8161 | 5460 |  | 8193 | 1659 |  | 9295 | 3140 |
| 1617 | 2034 |  | 8160 | 9332 |  | 6830 | 6244 |
| 5910 | 9428 |  | 8179 | 6415 |  | 1463 | 2158 |
| 9424 | 3360 |  | 9427 | 1202 |  | 8492 | 2160 |
| 8232 | 3215 |  | 5623 | 6312 |  | 6243 | 8211 |
| 8186 | 4617 |  | 9902 | 4608 |  | 2683 | 2605 |
| 9420 | 1304 |  | 6315 | 1811 |  | 8300 | 8371 |
| 9050 | 1710 |  | 3210 | 8116 |  | 8220 | 2981 |
| 4641 | 9325 |  | 9452 | 8496 |  | 4640 | 4599 |
| 9610 | 1726 |  | 8540 | 7600 |  | 9150 | 3220 |
| 5480 | 8280 |  | 1301 | 9433 |  | 8251 | 9495 |
| 2751 | 7289 |  | 2347 | 9429 |  | 8185 | 7650 |
| 6555 | 1720 |  | 9043 | 8030 |  | 8031 | 9900 |
| 7499 | 9184 |  | 5940 | 4624 |  | 1274 | 2703 |
| 6832 | 8120 |  | 6840 | 2704 |  | 9040 | 8190 |
| 5629 | 9181 |  | 9905 | 2691 |  | 1201 | 9620 |
| 8490 | 2075 |  | 8420 | 5972 |  | 1390 | 7291 |
| 6821 | 4626 |  | 9910 | 3101 |  | 6022 | 4606 |
| 7477 | 9426 |  | 9560 | 7620 |  | 9156 | 9409 |
| 8502 | 5622 |  | 7260 | 5833 |  | 9419 | 8034 |
| 4619 | 8480 |  | 5450 | 4598 |  | 8210 | 3019 |
| 3005.1 | 2303 |  | 4609 | 7380 |  | 2071 | 4604 |
| 9596 | 2688 |  | 8206 | 6020 |  | 7451 | 7501 |
| 3103 | 7461 |  | 8503 | 9230 |  | 7400 | 9322 |
| 6211 | 9403 |  | 9075 | 7660 |  | 9621 | 5260 |
| 5626 | 3350 |  | 1271 | 9590 |  | 7497 | 8499 |
| 9120 | 6200 |  | 7270 | 9927 |  | 8117 | 2661 |
| 6831 | 5700 |  | 7240 | 4603 |  | 6213 | 9594 |
| 8370 | 9623 |  | 6571 | 8510 |  | 9480 | 9300 |

Supplementary material 3. IAPS pictures number (Recognition task)

| Set 1 | |  | Set 2 | |  | Set 3 | |
| --- | --- | --- | --- | --- | --- | --- | --- |
| 2710 | 9623 |  | 8160 | 4603 |  | 1908 | 6825 |
| 2692 | 9321 |  | 1301 | 2691 |  | 1201 | 2661 |
| 7460 | 9490 |  | 8040 | 8116 |  | 9156 | 8211 |
| 3195 | 3360 |  | 8231 | 7492 |  | 8341 | 8190 |
| 6240 | 9325 |  | 7270 | 7380 |  | 9480 | 9430 |
| 6211 | 8370 |  | 6220 | 9433 |  | 6242 | 6834 |
| 8232 | 2120 |  | 9326 | 6250.2 |  | 9630 | 9570 |
| 3005.1 | 6021 |  | 9491 | 8531 |  | 8499 | 8034 |
| 9909 | 9425 |  | 9571 | 9904 |  | 5215 | 4604 |
| 2216 | 7461 |  | 1271 | 7450 |  | 1650 | 2155 |
| 9611 | 4625 |  | 9500 | 4608 |  | 9599 | 2208 |
| 8490 | 5628 |  | 8192 | 1310 |  | 6830 | 7650 |
| 6821 | 2034 |  | 2616 | 8501 |  | 6022 | 9594 |
| 1560 | 7405 |  | 2458 | 9423 |  | 8080 | 7570 |
| 9424 | 9184 |  | 6315 | 6212 |  | 6213 | 9302 |
| 9930 | 3350 |  | 8180 | 9622 |  | 9295 | 3140 |
| 8502 | 3215 |  | 8158 | 8496 |  | 8220 | 8065 |
| 3005.2 | 8500 |  | 5621 | 9911 |  | 6360 | 8200 |
| 7499 | 8041 |  | 9075 | 1811 |  | 7508 | 2071 |
| 8186 | 5700 |  | 5450 | 8178 |  | 9040 | 3220 |
| 9050 | 8400 |  | 8540 | 7660 |  | 8300 | 6244 |
| 5910 | 8163 |  | 9902 | 6312 |  | 9582 | 4606 |
| 6555 | 8280 |  | 8179 | 9230 |  | 6530 | 3019 |
| 9596 | 4626 |  | 6840 | 3101 |  | 8470 | 5260 |
| 9185 | 9426 |  | 6838 | 3185 |  | 7400 | 9300 |
| 4619 | 4617 |  | 8503 | 9590 |  | 4610 | 8467 |
| 8130 | 7515 |  | 8206 | 5972 |  | 6836 | 9495 |
| 5270 | 9903 |  | 8170 | 8208 |  | 9920 | 6410 |
| 4597 | 1019 |  | 9427 | 8250 |  | 8185 | 9160 |
| 2209 | 8480 |  | 4612 | 7600 |  | 7497 | 8090 |

Supplementary material 4. Valence and arousal rating of IAPS images

|  | | Set A | | Set B | | Set C | | *F* | *p*-value |
| --- | --- | --- | --- | --- | --- | --- | --- | --- | --- |
|  |  | Mean | SD | Mean | SD | Mean | SD |  |  |
| Encoding task | Positive |  |  |  |  |  |  |  |  |
|  | Valence | 6.87 | 0.70 | 6.87 | 0.69 | 6.86 | 0.67 | <.01 | .99 |
|  | Arousal | 5.64 | 0.59 | 5.64 | 0.66 | 5.64 | 0.65 | <.01 | .99 |
|  | Negative |  |  |  |  |  |  |  |  |
|  | Valence | 2.93 | 0.75 | 2.92 | 0.81 | 2.93 | 0.83 | <.01 | .99 |
|  | Arousal | 5.77 | 0.38 | 5.77 | 0.36 | 5.75 | 0.38 | .02 | .98 |
| Recognition task | Positive |  |  |  |  |  |  |  |  |
|  | Valence | 6.81 | 0.68 | 6.80 | 0.67 | 6.82 | 0.77 | .01 | .99 |
|  | Arousal | 5.65 | 0.56 | 5.78 | 0.70 | 5.73 | 0.59 | .35 | .71 |
|  | Negative |  |  |  |  |  |  |  |  |
|  | Valence | 2.85 | 0.66 | 2.85 | 0.78 | 2.86 | 0.64 | <.01 | .99 |
|  | Arousal | 5.78 | 0.36 | 5.82 | 0.30 | 5.79 | 0.38 | .13 | .88 |

Supplementary material 5. Comparing positive and negative images of valence and arousal rating

|  | | | Positive | | Negative | | *t*-value | *df* | *p*-value |
| --- | --- | --- | --- | --- | --- | --- | --- | --- | --- |
|  |  |  | Mean | SD | Mean | SD |  |  |  |
| Encoding task | Set A | Valence | 6.87 | 0.70 | 2.93 | 0.75 | 20.90 | 58.00 | <.001 |
|  |  | Arousal | 5.64 | 0.59 | 5.77 | 0.38 | −1.00 | 49.31 | .32 |
|  | Set B | Valence | 6.87 | 0.69 | 2.92 | 0.81 | 20.24 | 58.00 | <.001 |
|  |  | Arousal | 5.64 | 0.66 | 5.77 | 0.36 | –.95 | 44.92 | .35 |
|  | Set C | Valence | 6.86 | 0.67 | 2.93 | 0.83 | 20.30 | 58.00 | <.001 |
|  |  | Arousal | 5.64 | 0.65 | 5.75 | 0.38 | −.79 | 46.73 | .43 |
| Recognition task | Set A | Valence | 6.81 | 0.68 | 2.85 | 0.66 | 22.93 | 58.00 | <.001 |
|  |  | Arousal | 5.65 | 0.56 | 5.78 | 0.36 | −1.02 | 58.00 | .31 |
|  | Set B | Valence | 6.80 | 0.67 | 2.85 | 0.78 | 21.02 | 58.00 | <.001 |
|  |  | Arousal | 5.78 | 0.70 | 5.82 | 0.30 | −.27 | 39.62 | .79 |
|  | Set C | Valence | 6.82 | 0.77 | 2.86 | 0.64 | 21.63 | 58.00 | <.001 |
|  |  | Arousal | 5.73 | 0.59 | 5.79 | 0.38 | −.46 | 49.67 | .65 |

**
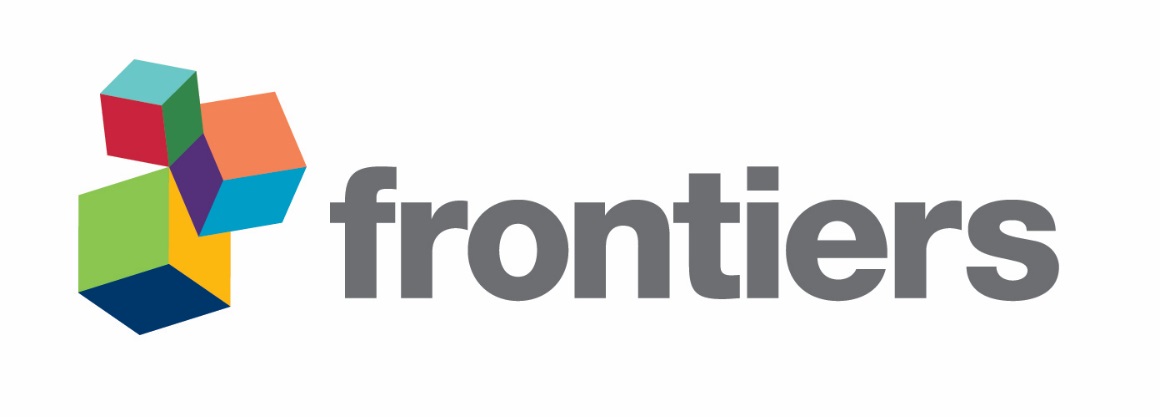
**
